# 

**Published:** 1882-11-20

**Authors:** 


### TABLE OF CONTENTS.

ORIGINAL AND SELECTED ARTICLES.

A Case in Practice, by G. A. Cook, M.D., of Georgia, 401. The Vomiting of Pregnancy, by Wm. B. Atkinson, M.D. 402. Note on the Delivery of the Placenta, by Charles Jewett, M.D., 406. Chinoidine and Capsicum in Intermittent Fever, by R. C. M. Page, M.D., New York, 408. The Climate of Florida, by James C. Neal, M.D., Ph. C., 411. Bronchocele Cured by the Hypodermic Injection of Tincture of Iodine, by O. W. Schindel, M. D., Cumberland, Md., 414. Dr. Reiter's Letter—Lappa Major, 415. The Chinese Druggist in New York, by Fred. Hohenthal, 417. Arsenic Internally and Subcutaneously in the Treatment of Lymphoma, 419.

ABSTRACTS AND GLEANINGS.

Extirpation of the Spleen, 420. Placental Delivery, 421. Cataract, Enucleation and Iridectomy, 422. Practical Notes on Neuralgia and its Treatment, 424. A New Sleep-producing Agent, 425. Nitrous Oxide, 426. Treatment of Diabetes by Bromide of Potassium; Recovery of Nine Cases of Hydrophobia, 427. Treatment of Intussusception; Treatment of Yellow Fever by Phenic Acid, 428. Viability of Premature Children; Poisoning from Red Stockings; Gum Album for Gastric Irritation and Headache, 429. Pet Animals and Contagious Diseases; Treatment of Syphilis; Small-pox and Scarlatina Occurring Simultaneously in the same Subject; Small-pox in Birds, 430.

SCIENTIFIC ITEMS.

The Ways of Plants; The London Times, 431. Cautious; Novel Use of the Electric Light; Mons. Camille Flammarion; The Transit of Venus, 432.

PRACTICAL NOTES AND FORMULÆ.

Torpor of the Colon; Remedy for Asthmatic Paroxysms; Dysmenorrhœa; 433. Nervine and Anti-Spasmodic; Apthous Sore Mouth in Infants; Treatment of Eczema of the Genitalia, Pruritus and Leucorrhœa, 434. Boracic Acid Ointment; A Specific for Singultus; Dr. Yuna's Vegetable Liver Pill, 435.

EDITORIALS AND MISCELLANEOUS.

Editorial Notes; Error; A Mad Doctor; Medical Progress, 436. A New York Doctor; Pamphlets Received, 437. Book Notices, 438. Receipted; Special Notices, 440.

